# Risk factors for visual field progression in newly diagnosed exfoliation glaucoma patients in Sweden

**DOI:** 10.1038/s41598-022-14962-9

**Published:** 2022-06-24

**Authors:** Marcelo Ayala

**Affiliations:** Eye Department, Skaraborg Hospital, Skövde, Sahlgrenska Academy, Gothenburg University & Karolinska Institute, 541 85 Skövde, Sweden

**Keywords:** Neuroscience, Health care, Risk factors

## Abstract

The present study aimed to identify risk factors for visual field progression in newly diagnosed exfoliation glaucoma patients. Prospective nonrandomized cohort study. The study included patients with newly diagnosed exfoliation glaucoma. All patients were followed for at least 3 years with reliable visual fields. Both risk factors at inclusion and during the 3-year follow-up were considered. For inclusion, five reliable visual fields were needed. Exfoliation glaucoma was defined based on the European Glaucoma Society guidelines. Visual field evaluation was performed using the 24–2 strategy of Humphrey field analysis. Outcomes: Visual field progression. Three different approaches were used: mean deviation, visual field index, and guided progression analysis. Independent variables were tested first in a univariate linear or regression model. The significant variables were retested in a multivariate linear or logistic regression model. The results were different for the MD, VFI and GPA models. The only variable that showed a significant association in the three models was age (*p* = 0.004; *p* = 0.006; *p* = 0.04). Significant variables in the two models were IOP at diagnosis (*p* = 0.02; *p* = 0.04), IOP reduction in absolute terms (*p* = 0.006; *p* = 0.003), IOP reduction in relative terms (%) (*p* = 0.04; *p* = 0.009) and number of medicines (*p* = 0.02; *p* = 0.002). Significant variables in one model were family history (*p* = 0.04), smoking (*p* = 0.03), cataract surgery (*p* = 0.04) and SLT treatment (*p* ≤ 0.001). Exfoliation glaucoma is a fast progressive glaucoma. Age at diagnosis must be considered. Significant IOP reduction must be achieved to slow down progress in exfoliation glaucoma. The use of SLT treatment should be advised in exfoliation glaucoma patients.

## Introduction

Glaucoma is an eye disease that affects the optic nerve. Usually, the visual fields are damaged, but in advanced cases, the disease can even lead to blindness. Together with age-related macular degeneration, it is one of the most common causes of blindness in Western countries^1^. A progressive loss of ganglion cells characterizes glaucoma. Unfortunately, no treatment has been discovered to cure the disease. The intrinsic causes of most glaucoma cases have not been found. However, several risk factors have been described. The most commonly cited risk factor for glaucoma development is increased intraocular pressure (IOP)^2^. There are several clinical manifestations of glaucoma; among them, the two most common clinical types in Sweden are primary open-angle glaucoma (POAG) and exfoliation glaucoma (EXFG)^3^.

Exfoliation glaucoma is secondary open-angle glaucoma characterized by protein-based material that deposits in the anterior chamber of the eye. The origin of these exfoliation deposits has not yet been discovered. Exfoliation has been isolated in different parts of the eye, but even other ocular and extraocular tissues showed the presence of exfoliation^4–7^. The deposits of exfoliation at the trabecular meshwork occlude the pores, diminishing the outflow of aqueous humor and thus increasing the IOP.

Risk factors for developing glaucoma have been well defined for POAG but not for EXFG. According to previous studies, risk factors commonly associated with POAG were age, elevated IOP at baseline, lower central corneal thickness, and family history^8–11^. It is supposed that similar risk factors can be applied for exfoliation glaucoma. However, genetic causes differed between POAG and EXFG^12^. Previous studies have also described risk factors for VF deterioration in POAG. In general, age and IOP peaks were defined as risk factors for visual field damage^8,10,11,13,14^, and exfoliation was even cited as a risk factor for progression^10,13^. Interestingly, no evidence was found about risk factors for visual field deterioration in newly diagnosed exfoliation glaucoma patients.

As described above, glaucoma is a progressive disease. Glaucoma progression is estimated by visual field deterioration. How progression develops varies significantly among individuals^15,16^. Therefore, it is essential to determine the rate of progression in every patient to choose the best treatment modality available. The European Glaucoma Guidelines^17^ recommend 5–6 visual fields 2 years after diagnosing glaucoma to estimate progression. The Swedish Glaucoma Guidelines^18^ recommend 5–6 visual fields in 3 years. This strategy was judged to be more realistic based on how the Swedish Health System works. Swedish patients' "gold standard" for glaucoma progression analysis is still visual field testing using computer-assisted perimetry.

The purpose of the present study was to identify risk factors for visual field progression in newly diagnosed exfoliation glaucoma patients. Both risk factors at diagnosis and risk factors during the 3-year control period were studied.

## Methods

The present study was a prospective nonrandomized cohort study. All patients with newly diagnosed exfoliation glaucoma were recruited. The inclusion period was from 1 January 2012 until 31 December 2016 (5 years). All patients attended the Ophthalmology Department at Skaraborg's Hospital. The Ophthalmology Department at Skaraborg's Hospital takes care of approximately 250 000 inhabitants.

### Inclusion criteria

Patients were suffering from newly diagnosed exfoliation glaucoma. The definition of exfoliation glaucoma was an untreated IOP of ≥ 21 mm Hg, open anterior chamber angle, glaucomatous visual field defect (at least two repeatable Humphrey 24–2), and glaucomatous optic nerve damage, all together with the presence of exfoliation material, according to the definition of the European Glaucoma Society^17^.

### Exclusion criteria

1. Patients who could not perform reliable visual fields at the beginning of the study and/or could perform at least five reliable visual fields 3 years after the exfoliation glaucoma diagnosis. The following criteria were used to define reliable visual fields: false-positives ≤ 15% and/or false negatives ≤ 20% and/or fixation losses ≤ 30%.
2. Patients with advanced glaucoma defined as a mean deviation (MD) ≥ 18 dB and/or visual field index (VFI) ≤ 40% were excluded. These patients were excluded to avoid "floor effects" in which further loss of visual field defects can no longer be detected^19,20^.
3. Patients needing glaucoma surgery during the follow-up period. Uncomplicated cataract surgery and/or selective laser treatment (SLT) were not considered as exclusion criteria.
4. Patients suffering from another eye disease could modify visual fields during the study period, such as central vein occlusion and retinal detachment.
5. Patients dropped out of the 3-year control period for different reasons, such as dementia and moving to another city.

The risk factors studied at diagnosis were age, sex, unilateral/bilateral glaucoma, visual acuity, refractive errors, IOP, central corneal thickness (CCT), gonioscopy: anterior chamber depth and pigmentation, cup-disc ratio (C/D), diabetes, smoking, hypertension, migraine and family history of glaucoma.

Risk factors studied under the 3 years were IOP reduction in absolute values, IOP reduction in relative values, SLT treatment, cataract operation, and the number of medications.

Every patient was examined in detail at inclusion. All patients were referred to the Ophthalmology Department due to high IOP (≥ 21 mmHg) detected by an optician/optometrist. Age was registered as age at glaucoma diagnosis. Sex was registered as male/female. The unilateral/bilateral presence of glaucoma was recorded. In the case that both eyes suffered glaucoma, one eye was chosen at random.

Visual acuity was measured using a Snellen chart. Refractive errors were registered based on the consultation chart sent by the optician/optometrist. For more straightforward calculations, the aspheric equivalent was calculated. The IOP was measured using a Goldman’s applanation tonometer. The central corneal thickness (CCT) was measured using an ultrasound device (Tomey Pachymetry; Tomey Corp, Nagoya 451–0051, Japan). The average value of seven measurements was automatically calculated. To assess the trabecular meshwork, gonioscopy was performed using goniolens with undilated pupils. According to Shaffer's system, the anterior chamber was studied and classified as deep (0–4). The trabecular meshwork was studied regarding pigmentation and ranked 0–3. The patient’s pupils were then dilated with 2.5% phenylephrine and tropicamide 0.5% (Bausch & Lomb UK Ltd, 106 London Road-Kingston-upon-Thames-Surrey-KT2 6TN-England). Eyes were classified as exfoliation if there was evidence of exfoliation material on the pupil, lens, or angle with dilated pupils. The status of the optic nerve was assessed using a 90-D lens, and the average vertical cupping was recorded as the cup-to-disc ratio (C/D).

Furthermore, all patients answered a questionnaire with questions regarding hypertension, smoking, diabetes, migraine, and family history of glaucoma. Hypertension was defined as "using medicines against high blood pressure" (Yes/No). Smoking was described as "smoking more than 50 cigarettes in your life" (Yes/No). Diabetes was defined as "using medicines against diabetes" (Yes/No). Migraine was defined as "suffered from migraine" (Yes/No). A family history of glaucoma was defined as having a near relative suffering from glaucoma. Near relatives were considered fathers and/or mothers and/or siblings. The answer was registered as "Yes/No."

Risk factors for progression under the 3 years were measured as follows: IOP reduction in absolute values was measured as the IOP 3 years after diagnosis (IOP at diagnosis-IOP at 3 years). The relative IOP reduction was calculated as a percentage (%).

The SLT treatments were measured as present or absent per patient. An uneventful cataract operation was not a reason for exclusion and was counted as present or absent. Eye drops at the end of the 3 years were measured as the number of medicines (compounds) and not as the number of bottles.

The endpoint of the study was visual field progression. All patients were examined using Humphrey field analysis (Carl Zeiss, Carl-Zeiss-Straße 22, 73,447 Oberkochen, Germany) using the software threshold 24–2. Three different methods for the assessment of visual field progression were used.

The first method was based on the "mean deviation" (MD) visual field parameter. The difference in MD values from the beginning to the end of the study was calculated. Higher values indicated higher progression. The MD values were chosen since several studies still use MD as an indicator of progression^16,21,22^. Cataract development can also modify MD values.

The second method used was glaucoma progression analysis based on the "visual field index" (VFI). The device calculated the VFI and performed a regression analysis calculating the "rate of progression" (ROP). The ROP was calculated automatically by the device as the amount of VFI deterioration (%)/year. The ROP calculation is also called a "trend analysis."

The third progression analysis used was guided progression analysis (GPA). The GPA is also included in the device and performed automatically (GPA Alert). The GPA is different from the previous one (ROP). The GPA alert analysis is called an "event analysis." The machine compares every single point to prior examinations. The GPA alert results can be no, possible or likely progression. For analysis purposes in this study, the results were evaluated as "no progression" or "progression," which included both "possible" and "likely" progression.

### Statistics

SPSS (IBM, 1 New Orchard Road Armonk, NY 10,504, USA) software was used for statistical analysis. The variables were tested in a “2-step manner”. In the first step, to test the association between variables and progression, univariate linear regression analysis was performed for continuous endpoint variables (MD and VFI). Then, for the dichotomous endpoint (GPA), a univariate logistic regression analysis was performed. The variables that showed a significant association in the univariate analysis were retested using multivariate analysis. Again, a multivariate linear regression analysis was used when the endpoints were continuous (MD and VFI); meanwhile, a multivariate logistic regression analysis was performed when the endpoint was dichotomous (GPA). The significance level was set at 0.05.

### Ethical approval

This study was performed in line with the principles of the Declaration of Helsinki. Approval was granted by the Ethics Committee of Gothenburg’s University (DN: 119–12.).

### Consent to participate

Informed consent was obtained from all individual participants included in the study.

## Results

In total, 91 patients were included in this study. Fourteen (n = 14) patients were excluded. The reasons for exclusion were n = 4 due to very damaged visual fields initially, n = 5 did not attend the check-up visits and/or could not perform reliable visual fields, n = 3 developed other eye diseases during the 3 years, and n = 2 had glaucoma operations. The average age at inclusion was 71.41 (± 7.52) years (range: 47–88). Regarding sex distribution, n = 49 (53.85%) were female, and n = 42 (46.15%) were male (chi-square; *p* = 0.46). Glaucoma was unilateral in n = 62 (68.13%) and bilateral in n = 29 (31.87%) (Chi-square; *p* =  < 0.001). At diagnosis, the average IOP value was 33.14 mmHg (± 6.66) (range: 22–55). Visual acuity at diagnosis was 0.82 (± 0.25). Refractive errors were 0.47 D (± 1.63), range: − 4.25/ + 3.25 D. The average CCT value was 541.6 µm (± 32.97). Gonioscopy at diagnosis showed an average anterior chamber depth of 3.13 (± 0.61), and pigmentation of the trabecular meshwork was 2.47 (± 0.64). The CD ratio at diagnosis was 0.73 (± 0.17).

Patients who reported hypertension were n = 50 (55%), while n = 41 (45%) reported no hypertension (chi-square; p = 0.34). Smoking was reported by n = 41 (45%), and no smoking was reported by n = 50 (55%) patients (chi-square; *p* = 0.34). Diabetes was reported by n = 14 (15.38%), while no diabetes was reported by n = 77 (84.62%) (Chi-square; *p* =  < 0.001). The presence of migraine was reported in only n = 6 (6.6%) cases, while n = 85 (93.4%) reported no migraine (Chi-square; *p* ≤ 0.001). A family history of glaucoma was reported by n = 39 (42.85%), while n = 52 (57.15%) reported no family history (chi-square, *p* = 0.17).

After 3 years, the average IOP was 17.88 mmHg (± 2.99). The absolute IOP reduction at 3 years was 15.35 mmHg (± 7.38). The relative IOP reduction at 3 years was an average of 47.28% (± 14.28). The number of cataract patients operated on during the 3-year follow-up period was n = 14. The number of patients treated with SLT was n = 16. All patients underwent 180° SLT treatment with approximately 50 spots. The spot size was fixed to 300 µm. The mean energy used per patient was 44 ± 2.3 mJ. No anti-inflammatory eye drops were used after SLT treatment. The number of medicines at the 3-year follow-up was 2.66 (± 0.89). Regarding the distribution of the medicines used, the most common was prostaglandin analogs (n = 83, 36.89%), followed by beta-blockers (n = 75, 33.34%), carbonic anhydrase inhibitors (n = 51, 22.67%) and alpha-agonists (n = 14, 6.22%). Pilocarpine eye drops were used only in n = 2 patients (0.88%).

Regarding visual field parameters at baseline, the MD was − 6.41 dB (± 5.01), and the VFI was 85.4% (± 14.64). After 3 years of follow-up, the visual field parameter was MD − 9.95 dB (± 6.31), and the VFI was 75.61% (± 19.12). The rate of progression based on VFI was − 2.60% (± 2.38)/year. The G.P.A. showed that n = 55 progressed and n = 36 did not progress (chi-square; *p* = 0.04).

The risk factors at diagnosis that showed a significant association with VF deterioration differed in the three models studied. In the MD model, the variables that showed a significant association were age, IOP reduction in absolute numbers, and cataract surgery during the follow-up period. Details are provided in Table 1.

**Table 1:** Significant variables tested in the univariate and the multivariate linear regression analysis using the MD difference as endpoint.

Variables	Univariate	Multivariate	
	*P* values	*P* values	β coef
Age	0.034*	0.004*	0.092
Smoking	0.005*	0.35	NA
Gonioscopy (Shaffer)	0.03*	0.14	NA
IOP diagnose	0.001*	0.30	NA
Cataract surgery	< 0.001*	0.04*	− 1.24
IOP reduction absolute	< 0.001*	0.003*	− 0.013
IOP reduction relative	< 0.001*	0.08	NA
Amount of medicines	0.023*	0.55	NA
SLT	< 0.001*	0.09	NA

(*) Significant values. *MD* Mean deviation; *IOP* Intraocular pressure; *SLT* Selective Laser Treatment; *NA* Non-applicable.

In the VFI model, the variables associated with progress were age, family history, smoking, IOP at diagnosis, IOP reduction (both the absolute and the relative values), the number of medicines, and SLT treatment. Details are provided in Table 2.

**Table 2:** Significant variables tested in the univariate and the multivariate linear regression analysis using the VFI (ROP) as endpoint.

Variables	Univariate	Multivariate	
	*P* values	*P* values	β coef
Age	0.004*	0.006*	0.053
Family history	0.02*	0.04*	0.129
Smoking	0.005*	0.03*	0.145
VA	0.026*	0.76	NA
Gonioscopy (Shaeffer)	0.034*	0.54	NA
IOP at diagnose	< 0.001*	0.02*	0.049
IOP reduction absolute values	< 0.001*	0.006*	− 0.070
IOP reduction relative values	0.025*	0.04*	− 0.022
Number of medicines	< 0.001*	0.02*	− 0.32
SLT	< 0.001*	< 0.001*	− 1.28

(*) Significant values. *VFI* Visual field index; *ROP* Rate of progression; *VA* Visual acuity; *IOP* Intraocular pressure; *SLT* Selective Laser Treatment; *NA* Non-applicable.

In the GPA model, the variables associated with progress were age, IOP at diagnosis, IOP reduction in relative values, and the number of medicines. Details are provided in Table 3.

**Table 3:** Significant variables tested in the univariate and the multivariate logistic regression analysis using the GPA (dichotomous: progress/no progress) as endpoint.

Variable	Univariate	Multivariate	
	*P* values	*P* values	Exp B
Age	0.02*	0.04*	1.12
Family history	0.03*	0.26	NA
Smoking	0.04*	0.76	NA
IOP diagnose	0.02*	0.04*	1.23
IOP reduction absolute	< 0.0001*	0.14	NA
IOP reduction relative	< 0.0001*	0.009*	1.07
Amount of medicines	< 0.0001*	0.002*	4.79

(*) Significant values. *GPA* Guided progression analysis; *VA* visual acuity; *IOP* Intraocular pressure; *NA* Non-applicable.

The only variable that showed a significant association in the three different models was age. The IOP at diagnosis, IOP reduction in absolute values, and IOP reduction in relative values showed a significant association in two of the three models studied. A family history of glaucoma, smoking, and SLT treatment showed a significant association only in the VFI model. A summary of the significantly associated variables was included for better visualization. Details are provided in Table 4.

**Table 4:** Summary of the significant variables tested in the multivariate analysis according to the endpoints.

	MD	VFI	GPA
Age	X	X	X
Family History		X	
Smoking		X	
Cataract surgery	X		
IOP at diagnose		X	X
IOP reduction absolute	X	X	
IOP reduction relative		X	X
Amount of medicines		X	X
SLT		X	

*MD* Mean deviation; *VFI* Visual field index; *GPA* Guided progression analysis; *IOP* Intraocular pressure.

## Discussion

The present study tried to elucidate the risk factors for visual field deterioration in a cohort of newly diagnosed exfoliation glaucoma patients in Sweden. The risk factors studied were recorded both at diagnosis and during the 3-year follow-up period.

Previous studies have extensively described risk factors for developing glaucoma^8–11^. In addition, risk factors for progression in glaucoma damage have also been described before^23^. Interestingly, exfoliation seems to be a risk factor for fast progression in previous studies compared with other glaucoma types. However, evidence about whether the described risk factors apply for newly diagnosed exfoliation glaucoma patients is lacking. In several previously published studies, exfoliation glaucoma patients were excluded due to the low prevalence of exfoliation glaucoma in other populations and because the disease develops differently. Exfoliation glaucoma patients showed a more aggressive and rapid development of visual field damage than primary-open glaucoma patients^24^. At our department, approximately 60% of all newly diagnosed glaucoma patients suffer from exfoliation glaucoma, making the results from this study very interesting from a clinical point of view.

Age was shown to be a common risk factor for progression in the three models studied. The average age at inclusion was 71.41 (± 7.52) years. This result agrees with previous studies in which the average age at glaucoma diagnosis was approximately 70 years old^25^. Only three patients were excluded from the study due to their inability to perform visual field analysis. All of them were approximately 70 years old, meaning that no patient was indirectly excluded due to age. Considering the β coefficients in the multivariate linear regression analysis, the MD values increased by 0.092 dB/year. The MD increase can be due to age (cataract) or glaucoma progression. However, age was also positively associated with visual field progress when considering the rate of progression (ROP). ROP is based on the visual field index (VFI), which corrects for cataracts and evaluates more accurate glaucoma damage. The β coefficient in the multivariate linear regression analysis showed an ROP deterioration of 0.053%/year. This increased ROP value means that a patient diagnosed with glaucoma at 80 years old had faster visual field deterioration than a patient diagnosed at 70 years old. The average ROP was 2.60%/year for the whole cohort. In the hypothetical case in which a patient aged 70 progressed at 2.60%/year, a patient aged 80 would progress at 2.65%/year. These results must be interpreted with caution. The results apply to the range studied, which was 47–88 years.

Furthermore, the model was linear; this assumption can be suitable for the years studied but probably not for older ages. Other models, such as exponential models, are probably more realistic at older ages. Further studies are needed to clarify the best models for different age groups. The study showed increased progress with increased age. The increased progress with age could be because the number of ganglion cells diminished with age, so the visual fields deteriorated faster as age increased.

Several previous studies described IOP as the most critical risk factor for glaucoma development. The present study showed an association between IOP at diagnosis, IOP reduction in absolute and relative values, and VF deterioration in newly diagnosed exfoliation glaucoma patients. The average IOP was high at diagnosis, 33.14 mmHg (± 6.66). Exfoliation glaucoma is a high IOP glaucoma. Exfoliation material obstructs the trabecular meshwork, producing high IOP levels. The average ROP in the cohort was 2.60%/year during the 3-year follow-up. Based on the results from the multivariate analysis for ROP (β coefficient), every mmHg IOP at baseline represented an increased ROP of 0.05. Theoretically, this means that a patient with a baseline IOP of 25 mmHg and an ROP of 2.60%/year would progress at 2.65% year if the IOP was 35 mmHg or at 2.70%/year if the IOP was 45 mmHg. As pointed out above, this extrapolation must be taken with caution. This probably applies to the IOP levels registered in this study (22–55 mmHg) and probably not for other IOP levels.

Furthermore, the model assumed that the relationship between IOP values and ROP was linear. This is not known; each mmHg probably has a different impact on ROP if the IOP is approximately 30 mmHg than if it is approximately 50 mmHg. Further studies are needed to clarify this issue.

IOP reduction is another interesting issue to discuss. The present study included both absolute and relative values for IOP reduction after diagnosis. Patients included in this study were followed and treated according to our guidelines for the treatment and follow-up of glaucoma patients. All patients reached “target IOP” after diagnosis. This “target IOP” is an uncertain IOP value that is usually approximately 18–20 mmHg, and it was established by an ophthalmologist who made the diagnosis. According to our guidelines, 3 years after follow-up with at least 5–6 reliable visual fields, this "target IOP" must be re-evaluated. In the present study, the IOP 3 years after follow-up was 17.88 mmHg (± 2.99). Even if this value can be considered acceptable, 2/3 of patients showed progression in the GPA analysis. Therefore, it seems that the "target IOP" should be lower than 18 mmHg, probably approximately 16 mmHg (or even lower), to diminish the visual field's deterioration. A similar approach can be made with regard to the relative IOP reduction. A common assumption is that a 20% IOP reduction is enough to stop the progression of the disease^26^. In the present study, the average IOP reduction was 47.28% (± 14.28). However, 2/3 of glaucoma patients showed progression according to the GPA analysis. The aggressive nature of these newly diagnosed exfoliation glaucoma patients probably requires a higher relative IOP reduction to stop the progression of the disease.

The absolute IOP reduction was significantly associated with MD and VFI but not with the GPA model. Meanwhile, the relative IOP reduction was significantly associated with the VFI and the GPA but not the MD model. This means that both ways to control IOP reduction are needed. However, in our clinical practice, we usually forget the importance of considering both absolute and relative IOP reduction. We usually focus more on the absolute values, and sometimes we feel that it is difficult to find the IOP value at diagnosis and calculate the relative IOP reduction. The present study showed the importance of checking both values to establish a more accurate level of IOP that can stop VF deterioration.

Three variables showed a significant correlation with visual field progression only in the VFI model. The mentioned variables were family history, smoking, and SLT. Family history of glaucoma was measured in the present study as present or absent. Only the closest relatives were considered: mother and/or father and/or siblings. The information was self-reported, and reporting bias might be admitted. A family history of glaucoma was reported by n = 39 (42.85%), while n = 52 (57.15%) reported no family history (chi-square, *p* = 0.17). Most of the patients were born in Sweden, and their parents were also born in Sweden. Only two patients had Finnish parents. Genetic mechanisms behind exfoliation glaucoma have been described in previous studies^27^. However, no previous evidence regarding family history and a poorer prognosis for glaucoma has been found.

Smoking has been described as a risk factor for glaucoma development^28^. In the present study, smoking was measured as present or absent based on the following question: "Did you smoke more than 50 cigarettes in your life?” Additionally, this question was self-reported, addressing the possibility of reporting bias. Interestingly, a quite high number of patients reported that they had smoked (n = 41; 45%). Smoking is not so common among the new generations in Sweden. Furthermore, the study did not ask if the patients took “snuff” (chewing moist powder tobacco), which is common in Sweden. The increased visual field deterioration among smokers can be explained by decreased blood circulation in small vessels due to nicotinamide effects.

The SLT was also recorded as present or absent to facilitate the analysis. Interestingly, the association was negative; patients treated with SLT showed less progression according to the VFI model. The decision to treat with SLT was based on the clinicians who met the patients. SLT treatment has shown promising results in exfoliation glaucoma patients^29^. However, the number of patients treated with SLT in the present study was relatively low (n = 16). Based on the current study results, it is possible to recommend SLT to exfoliation glaucoma patients.

The number of medicines was also associated with visual field progression in the present study. In the VFI model, the association was negative; an increased number of medicines rendered a lower progression. At 3 years of follow-up, the patients were treated with 2.66 (± 0.89) drugs. Based on the β coefficient of the multivariate linear regression, each medicine reduced the ROP by 0.32%/year. The higher number of drugs induced a higher reduction in the IOP, thus diminishing progression. It is also possible that medications can reduce progression by mechanisms other than IOP reduction (neuroprotection).

The relationship between the number of medicines and progression was also demonstrated in the GPA (dichotomous) model. The logistic regression analysis showed a significant association (*p* = 0.002) between the number of medications and the detection of progress/no progress of the visual fields in the GPA. A post hoc analysis showed that the average amount of medicines in the no progression group was n = 2.1 ± 0.8; meanwhile, in the progression group, n = 3 ± 0.7. The estimated odds ratio (OR) coming from the regression analysis was 4.79. This means an increased risk of being treated with more medicines if you are suffering from progressing glaucoma than if your glaucoma is not progressing. A post hoc analysis was performed to clarify the issue. The patients were divided into Groups 1: 1 or 2 medicines and Group 2: 3 or 4 (only one patient was treated with five medications and was excluded). Then, an OR was measured between the two groups, showing a value of 8.47. Therefore, individuals with progressive glaucoma are eight times more likely to be treated with 3 or 4 medicines than individuals who suffer from nonprogressive glaucoma. This is quite logical during the follow-up period if the ophthalmologist suspected progression and wanted to decrease IOP even more. Additionally, it must be considered that high IOP at diagnosis will increase progression itself and require an increased number of medications.

Cataract surgery was performed in 14 patients during the 3-year follow-up period. At baseline, 23 patients had already undergone cataract surgery. Interestingly, cataract surgery showed an association with progression only in the MD-based model. Cataract surgery improved the MD values in the visual fields. The improvement of visual fields due to cataract surgery was not observed in the two other models (VFI and GPA). Currently, there is a general agreement not to use MD to evaluate glaucoma progression since cataract surgery can alter the parameter. MD was included in the present study because it has been included in several previous studies^25^. The other reason is that the most common glaucoma classification (Hodapp’s classification) is MD values^30^. The included patients in this study mainly belonged to the early and moderate glaucoma groups.

The study has certain limitations. As noted above, most patients belonged to the early (MD = 0–5.9 dB) and moderate (MD = 6–12 dB) glaucoma groups. Very few patients with advanced glaucoma were included. Patients were excluded if they showed an MD ≥ 18 dB and/or VFI ≤ 40%. This criterion was chosen to avoid "floor effects” in which further loss of visual field defects can no longer be detected^19,20^. A significant limitation of the study is the exclusion of very advanced glaucoma subjects (in the study, patients were excluded if they showed an MD ≥ 18 dB and/or VFI ≤ 40%). Unfortunately, advanced glaucoma patients cannot be followed with visual fields. Progression assessment would not be accurate when the visual fields were so damaged.

Another limitation of the study is that no morphological assessment of the optic nerve was performed. The only parameter included was the cup/disc ratio (C/D), but this is a subjective manner of optic nerve assessment. In addition, the use of optic nerve OCT was not as common when the study began. Currently, OCT technology is one of the tools for glaucoma follow-up at our department. However, it seems that OCT also has "floor effects" for advanced glaucoma patients^31^. Nevertheless, there is no consensus about whether morphology or function is the best way to evaluate glaucoma progression^32^.

A possible strength of the present study is that all included patients were naïve or newly diagnosed exfoliation glaucoma patients. There is no information available in the literature about this special patient group and how glaucoma develops. Furthermore, the number of patients included (n = 91) rendered the results reliable and of clinical importance.

In conclusion, the present study showed that age was the most critical risk factor for progression in newly diagnosed exfoliation glaucoma patients in Sweden. The other factors to be considered are IOP at diagnosis and IOP reduction in both absolute and relative numbers. Factors with a lower impact on progression were positive family history, smoking, and SLT. Despite a lower impact on progression, SLT treatment and stopping smoking should be recommended in this patient group to slow progression. Exfoliation glaucoma is an aggressive form of glaucoma that must be monitored often to avoid advanced visual field damage and blindness.

## Data Availability

The datasets used and/or analysed during the current study available from the corresponding author on reasonable request.
